# Spermidine Induces Expression of Stress Associated Proteins (SAPs) Genes and Protects Rice Seed from Heat Stress-Induced Damage during Grain-Filling

**DOI:** 10.3390/antiox10101544

**Published:** 2021-09-28

**Authors:** Min Chen, Yuying Fu, Qingshan Mou, Jianyu An, Xiaobo Zhu, Temoor Ahmed, Sheng Zhang, Farwa Basit, Jin Hu, Yajing Guan

**Affiliations:** 1 Institute of Crop Sciences, College of Agriculture and Biotechnology, Zhejiang University, Hangzhou 310058, China; 11616036@zju.edu.cn (M.C.); 22016181@zju.edu.cn (Q.M.); anjy@zju.edu.cn (J.A.); 11916138@zju.edu.cn (F.B.); jhu@zju.edu.cn (J.H.); 2 Institute of Horticulture, Anhui Academy of Agricultural Sciences, Hefei 230041, China; 11716011@zju.edu.cn; 3 Hainan Research Institute, Zhejiang University, Sanya 572025, China; 22016139@zju.edu.cn; 4 State Key Laboratory of Rice Biology, Institute of Biotechnology, Zhejiang University, Hangzhou 310058, China; temoorahmed@zju.edu.cn; 5 Taizhou Agricultural Technology Extension Center, Taizhou 318000, China; hecan666@zju.edu.cn

**Keywords:** high-temperature, spermidine, seed quality, stress-associated proteins, thermotolerance

## Abstract

Heat stress during seed maturation significantly reduced seed size and quality. Polyamines, especially spermidine (Spd), were reported to be closely related to seed development and plant heat tolerance. Stress-associated proteins (SAPs) also played a critical role in plant heat resistance, but the relationship between Spd and SAPs in improving rice tolerance to heat stress during grain filling has not been reported. Our results showed that the external spraying Spd (1.5 mM) significantly increased seed germination rate, germination index, vigor index and 1000-grain weight, significantly increased endogenous Spd, spermine (Spm) content and peroxidase activity; significantly reduced MDA content; and greatly alleviated the impact of heat stress on rice seed quality during grain filling stage as compared with high temperature control. *OsSAP5* was the most upregulated expression induced by Spd, and may be mainly involved in the Spd-mediated enhancement of high-temperature resistance during rice seed development. Overexpression of *OsSAP5* in *Arabidopsis* enhanced 1000-grain weight and seed heat resistance. Exogenous Spd alleviated the survival rate and seedling length, reduced MDA content, and upregulated the expression levels of *SPDS* and *SPMS* in *Atsap4* mutant under high temperature during seed germination. In all, exogenous Spd alleviated the heat damage on seed quality during the grain filling stage and seed germination stage by improving endogenous Spd and Spm. *OsSAP5*, a key gene induced by Spd, might be involved in the rice heat resistance and seed quality in coordination with Spd and Spm.

## 1. Introduction

Increasing global temperature has become a significant constraint on global agricultural sustainable development in recent years. Rice (*Oryza sativa* L.) is one of the most important crops in the world, but it was sensitive to hot temperatures, especially at the heading and filling stages [1,2]. Pollen viability was greatly affected by heat stress, resulting in low seed setting rate and yield decline [3]. Meanwhile, the period of grain development was shortened under heat stress, resulting in insufficient grain filling and nutrition accumulation decreased seed size, and low seed quality [4,5]. It has been proved that for every 1 °C increase in the minimum temperature during the growing season, rice grain yield decreases by 10% [6]. However, the effect of heat stress during grain filling on offspring germination and development remains to be elucidated.

Polyamines (PA), including putrescine (Put), spermidine (Spd), and spermine (Spm), are a class of significant physiologically active substances in plants, involved in plant growth and development and response to various abiotic stresses, among which Spd is closely related to seed development and plant heat tolerance [7,8,9,10,11]. Soaking seeds in Spd significantly enhanced seed vigor, while exogenous cyclohexylamine (CHA), a Spd synthesis inhibitor, significantly inhibited seed germination and reduced seed vigor [12]. Some studies had found that the contents of Spd and Spm in rice were higher in well-developed grains [13]. The endogenous PAs contents in heat-resistant rice varieties were more stable than those in heat-sensitive ones under high-temperature stress. Changes in seed size and weight of sweet corn were positively correlated with endogenous Spd [14]. In *Arabidopsis*, dual mutants encoding the Spd synthesis genes *AtSPDS1* and *AtSPDS2* were shown a sharp decrease in endogenous Spd content, and seed development was severely impeded [15]. RNAi-mediated silencing of spermidine synthase gene resulted in decreased pollen viability and seed setting rate delayed seed germination and decreased germination rate of tobacco [16]. In addition, studies had shown that exogenous Spd enhances the heat resistance of rice seeds by regulating endogenous starch and polyamine metabolism [7], and significantly reduced the damage caused by high-temperature stress at the wheat filling stage [9] and lettuce seedling stage [17]. However, how Spd regulates grain filling in rice under high-temperature stress during seed development remains unclear.

Stress-associated proteins (SAPs) are novel A20/AN1 zinc finger domain proteins that are widely involved in abiotic stress. SAPs had been well studied in animals, but their roles and mechanisms in plants are still unclear. SAPs are prevalent in many species, including *Oryza sativa* (18 family genes), *Arabidopsis thaliana* (14 family genes), and *Brassica napus* (57 family genes) [18,19], and can be induced by one or more kinds of stresses. For instance, *OsSAP1* was the first *SAP* gene found in plants that could be induced by low temperature, drought, salt, heavy metals, and abscisic acid (ABA). Overexpression of rice *OsSAP1* in tobacco increased the tolerance of tobacco to cold, dehydration, and salt stress [20]. In addition, the expressions of *OsSAP1, OsSAP9, OsSAP11, OsSAP12, OsSAP14,* and *OsSAP17* were upregulated under cold stress [21]. The gene *ZFP177* (*OsSAP9*) identified in japonica rice responds to both cold and heat stress and was down-induced by salt stress [18]. AtSAP5 and MBF1c jointly promote heat stress tolerance in *Arabidopsis thaliana* [22]. Expression of *AtSAP5* in cotton upregulated putative stress-responsive genes and improved the tolerance to rapidly developing water deficit and moderate heat stress [21].

Previous studies had found that SAPs improved plant stress resistance by accelerating the accumulation of nitric oxide (NO) [23]; regulating transcription factors (TFs) such as MYC binding protein (MBP), dehydration-responsive element-binding protein (DREB), and DREB2A-interacting protein (DRIP); increasing antioxidants enzyme activity; and interacting with heat shock protein (HSP) [21,24,25,26]. At the same time, PAs can interact with hydrogen peroxide (H_2_O_2_), NO, ABA, or TFs such as heat shock factor (HSF) and other signaling molecules in response to plant stresses [8,27,28,29,30,31]. It shows that both PAs and SAPs can improve the stress tolerance of plants through stress-related transcription factors, but whether there is an interaction between PAs and SAPs to improve the heat resistance of plants has not been reported.

## 2. Materials and Methods

### 2.1. High-Temperature Treatment during Rice Development

The male and female seeds of hybrid Indica rice (*Oryza sativa* L. ssp. *Indica*) “Yliangyou 689” used in this experiment were obtained from the Zhejiang Agricultural Science Seed Company (Hangzhou, China). The plants of “Yliangyou 689” were grown on the test field of Zhejiang University under normal conditions with unified management. At 3 days after pollination (DAP) during the rice filling stage, each spikelet was sprayed with 4 mL of 1.5 mM Spd daily for three days (from 3 DAP to 5 DAP). Spraying of 4 mL of 20 mM CHA and 4 mL of distilled water (H_2_O) were respectively used as a negative control and normal control. At 5 DAP, all treated plants were put into high-temperature growth chambers for 5 days (40 °C 12 h light/35 °C 12 h dark, 60% relative humidity) and then transferred to the normal condition (26 °C 12 h light/21 °C 12 h dark, 60% relative humidity) until seed maturation. Meanwhile, the spikelets sprayed with H_2_O under normal growth conditions were also used as a control. To study the effect of Spd treatment on mature seed quality, all seeds were harvested at their maturity stage (35 DAP).

### 2.2. OsSAP5 Genetic Transformation

The ortholog of *OsSAP5* (LOC_Os02g32840) in *Arabidopsis* (Columbia-0, Col-0), *AtSAP4* (AT2G36320) was obtained from the TAIR website, and its mutants (SALK_152010 and SALK_030732) were purchased from the *Arabidopsis* Biological Resource Center (ABRC, Columbus, OH, USA) (https://abrc.osu.edu/researchers/, accessed on 31 July 2003 and 19 December 2001, respectively). It needs illustration was that *atsap4#5-1* and *atsap4#7-1* correspond to SALK_52010C and SALK_30732C. Meanwhile, genetic transformation of *OsSAP5* was performed in *Arabidopsis thaliana*; 465 bp of the ORF of *OsSAP5* was amplified by PCR, digested with EcoR I and Hind III, and cloned into pCAMBIA1301 vector using primers (1) and (2) of *OsSAP5* as follows: 

Forward primer 5′-CGCGATCCATGGCTGAGGAGCAGAGGTG-3′ (1)

Reverse primer 5′-AAAACTGCAGGATCTTGTCCTTGAGCTTGT-3′ (2)

The pCAMBIA1301 vector was subsequently recombined into pMD19-T using the T4 DNA Ligase. The binary vector was introduced into agrobacterium strain GV3101 and Col-0 plants were transfected through the floral dip method [32]. *OE*-*OsSAP5* transformed seeds were selected by hygromycin (HYG) on half-strength Murashige and Skoog (MS) medium (containing 1.2% sucrose and 0.6% agar, pH adjusted to 5.7). The *AtSAP4* mutants and *OE*-*OsSAP5* seeds of *Arabidopsis* were screened homozygously to T3 generation.

### 2.3. Function Verification of OsSAP5 and Spd

The *AtSAP4* mutants and *OE*-*OsSAP5* seeds of *Arabidopsis* were used for the following experiments. 

High temperature during seed germination stage: seeds were surface sterilized with 75% ethanol for 1 min and then with 0.01% sodium hypochlorite solutions for 20 min, and subsequently washed with sterilized water. All the seeds were grown directly on half-strength MS medium, stratified at 4°C for 4 days and then transferred to a standard plant incubator with the following settings: HT (high-temperature treatment, 50 °C), NT (normal temperature treatment, 23 °C) and SPD + HT (50 °C high-temperature treatment, 1mM Spd is added to the half-strength MS medium) for another 7 h in dark. After heat treatment, the *Arabidopsis* was moved to normal conditions (23 °C and 16/8 h day/night) for 7 days.

High temperature during grain filling stage: *Arabidopsis thaliana* grew normally (23 °C and 16/8 h day/night) until flowering, and the non-flowering plants were removed 3 days after full-blossom and placed in darkness at 35 °C for 4 h (HT), compared with the control at 23 °C (NT). After treatment, they were grown under normal conditions until harvest.

### 2.4. Seed Germination Assay 

The seeds of different treatments sampled at each harvest time were tested by the seed standard germination (Table 1). Each treatment had 3 replicates with 100 seeds per replicate. The seeds were placed on moist germinating paper and incubated in a growth chamber for 14 days under 30 °C 8 h light /30 °C 16 h dark. Germinated seeds (defined as the seed radicle visibly protruding through the seed coat and reaching half the length of the whole seed) were counted daily. The germination percentage (GP) was calculated on the 14th day; meanwhile, shoot height (SH) was measured, and the seedling dry weight (SDW) was determined after drying at 80 °C for 24 h. The above measurements were based on ten randomly selected normal seedlings for each replication. In addition, the germination index (GI) was calculated according to GI = Σ (Gt/Dt), where Gt is the number of germinated seeds per day, Dt is the time corresponding to Gt in days, and Dt is the number of germination days. Seed vigor index (VI) was determined according to the formula VI = GI × SDW [33].

### 2.5. Polyamines Measurement

The concentrations of PAs were measured according to the method of Gao [34] with slight modification. Briefly, 0.2 g of fresh seeds with treatments was homogenized with 2 mL of 5% (*w*/*v*) cold HClO_4_. Then the seeds were incubated in ice for 1 h, centrifuged at 12,000 rpm for 30 min at 4 °C, and then the supernatant was stored at −80 °C for PAs measurements. Thereafter, 1 mL of 2 mM NaOH and 10 μL of benzoyl chloride were added to 0.5 mL of the obtained supernatant and the mixture was then vortex-mixed vigorously and incubated in 37 °C water for 20 min. Then, 2 mL of saturated NaCl solution and 2 mL of diethylether were added to the mixture. After 1500× *g* centrifugation for 5 min at 4 °C, 1 mL of the ether phase was obtained and evaporated with a warm air-stream. The dried materials were dissolved in 100 μL methanol and its filtration through a 0.22 μm filter was subjected to reading on an HPLC, which included a 45 mm × 250 mm, 5 μm particle size reverse-phase (C18) column (Waters Nova-Pak), and a Waters 2487 dual λ absorbance detector. The mobile phases consisted of methanol-water (64:36, *v*/*v*) at a flow rate of 1.0 mL/min. Three PAs standard samples of Put, Spd, and Spm were prepared at different concentrations for the development of standard curves.

### 2.6. Malondialdehyde Content

Malondialdehyde (MDA) content was determined as 2-thiobarbituric acid (TBA) reactive metabolites [33]. Rice seed samples (0.3 g) were taken per treatment and homogenized in 8 mL of 50 mM potassium phosphate buffer (pH 7.8) under ice-cold conditions. Approximately 1.5 mL extract was homogenized in 2.5 mL of 5% TBA made in 5% trichloroacetic acid (TCA). The mixture was heated at 100 °C for 15 min, and then quickly cooled on ice. After centrifugation at 1800× *g* for 10 min, the absorbance of the supernatant was measured at 532 nm. Correction of nonspecific turbidity was made by subtracting the absorbance value measured at 600 nm. The concentration of MDA was calculated in terms of nmol mg^−1^ protein.

### 2.7. RNA Isolation and qRT-PCR

Frozen seeds (100 mg) were ground thoroughly in liquid nitrogen using a pestle and mortar by using RNA isolation (Takara, Japan) following the manufacturer’s instructions. The concentration and purity of the RNA were determined by NanoDrop 2000/2000c (Thermo Scientific, Agawam, MA, USA). A PrimerScript^TM^ RT reagent Kit (Transgen, Beijing, China) was used for RNA reverse transcription, and quantitative real-time RT-PCR was performed using SYBR premix EX Taq (Takara, Shiga, Japan). Rice housekeeping gene *OsActin* was used as internal reference to normalize the expression levels. The expression level was calculated using the 2^−∆∆CT^ method. There are three biological replicates in each experiment. All the primers used are listed in Table S1.

### 2.8. Statistical Analysis

The data were subjected to an analysis of variance (ANOVA) on the Statistical Analysis System (SAS) software 9.2. The multiple comparisons for mean values were performed by the least significant difference (LSD) test (α = 0.05). Before ANOVA, the data were transformed according to y = arcsin [sqrt (x/100)].

## 3. Results

### 3.1. Effects of Exogenous Spermidine on Rice Seed Quality under High Temperature during Early Seed Filling Stage

When the rice was subjected to high temperature (HT) stress during the early stage of grain filling (from 6 to 11 DAP), the germination percentage (GP), germination index (GI), vigor index (VI), and 1000-grain weight of mature seeds significantly reduced compared with the normal temperature (NT) (Table 1). However, the reduction could be significantly alleviated by exogenous Spd spraying, especially the GP and GI reached a similar level as the NT, while the CHA treatment further reduced seed quality as compared with the water spraying under HT (Table 1).

**Figure 1:**
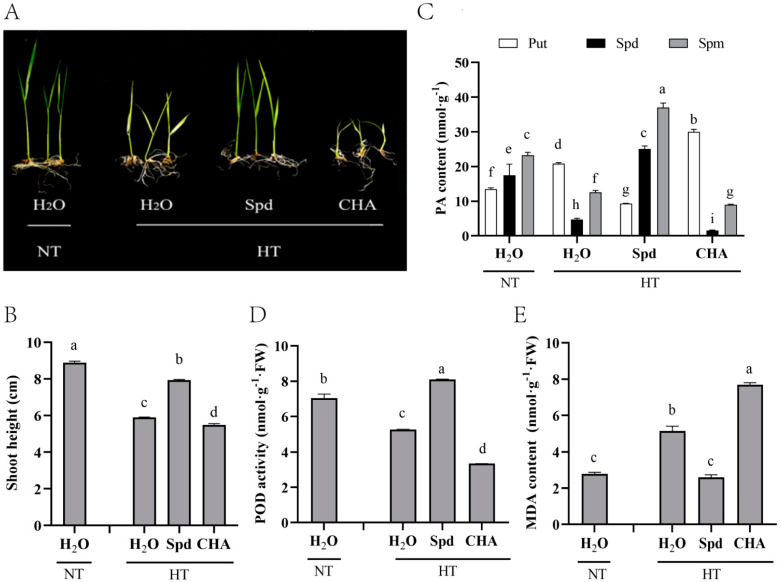
Effects of Spd spraying after pollination on seed endogenous polyamines and seedling quality of mature rice which experienced high temperature during the early grain-filling stage. (**A**) Seedling growth of mature rice harvested at 35 days after pollination. (**B**) Seedling shoot height of mature rice harvested at 35 days after pollination. (**C**) Endogenous polyamines (PA) content. (**D**) peroxidase (POD) activity and (**E**) malondialdehyde (MDA) content in seeds that experienced 5 days of high temperatures and were harvested 11 days after pollination. Spermidine (Spd), cyclohexylamine (CHA), putrescine (Put), spermine (Spm). Four biological replicates for each treatment were set. The diverse lowercase(s) on top of the bars were indicative of significant differences (LSD, α = 0.05) across treatments. HT meant high temperature (40 °C 12 h light/35 °C 12 h dark), NT meant normal temperature (26 °C 12 h light/21 °C 12 h dark).

After exogenous Spd spraying, the endogenous content of Spd and Spm in mature rice seeds significantly increased when subjected to HT for 5 days during early seed development. CHA significantly decreased the seed Spd and Spm contents as compared with Spd treatment and the water spraying controls under HT and NT. The changing trend of Put content was just the opposite (Figure 1C). In addition, Spd treatment significantly increased the shoot height, peroxidase (POD) activity, and decreased MDA content of seedlings as compared with the control under HT. By contrast, the CHA severely inhibited seedling growth and shoot height (Figure 1B–D). 

### 3.2. Expression Analysis of SAP Gene Family in Rice Treated by Spd under High Temperature during the Grain Early Filling Stage 

The *OsSAP* genes were mainly involved in abiotic stresses such as high temperature, drought, low temperature, and salt in rice [20,35,36,37]. It was reported that *AtSAP5* played a role in heat-responsive gene regulation together with MBF1c [22]. To determine whether *OsSAPs* is related to high temperature stress, we analyzed the response of the *OsSAP* gene family to high-temperature and exogenous SPD treatment during the filling period.

**Figure 2:**
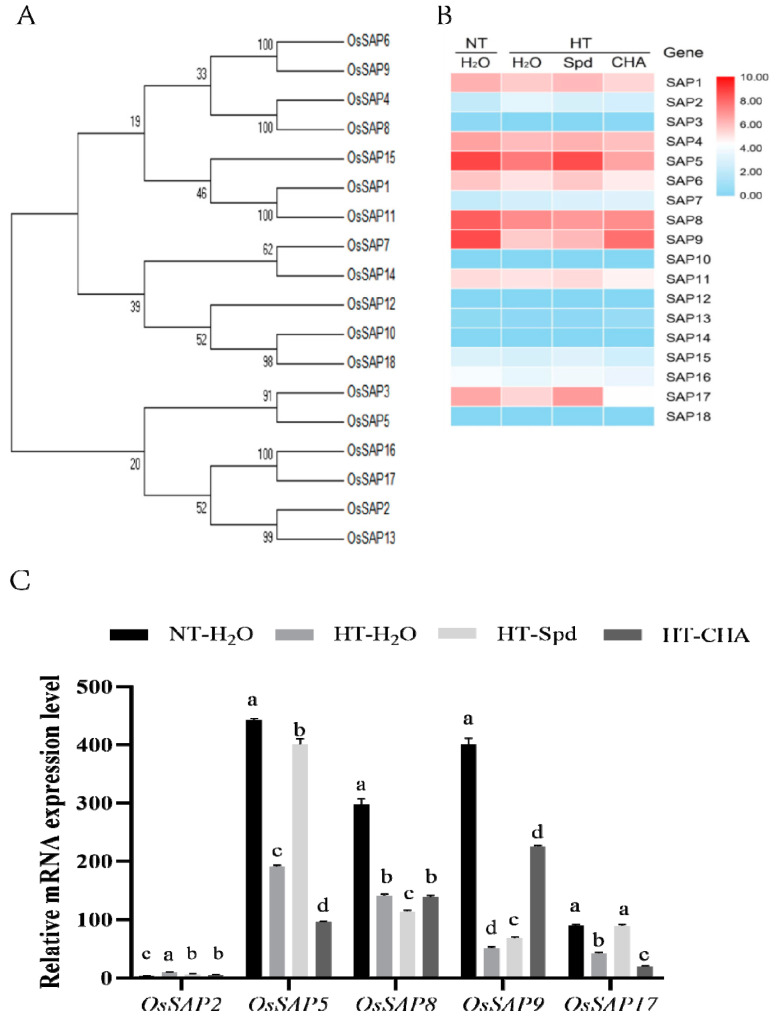
Statistics of expressed genes of *OsSAPs* family during early filling stage of rice. (**A**) Systematic evolution analysis of *OsSAP* family members. (**B**) Expression kinetics of 18 *OsSAP* family genes in response to heat stress during early seed filling stage (the seeds underwent 5 days of high temperatures and were harvested 11 days after pollination). (**C**) Relative mRNA expression level of *OsSAP2, OsSAP5, OsSAP8, OsSAP9* and *OsSAP17* during early seed filling stage (the seeds underwent 5 days of high temperatures and were harvested 11 days after pollination). The heatmap was generated by R package (pheatmap) using normalized (separate z-score computed per gene) expression values in units of qRT-PCR. The Illustrator software CS6 was used for creating the picture. The gene levels from low (0.00) to high (10.00) indicated the lowest and highest levels in the whole database. Real-time quantitative PCR was performed using three biological replications, and each was made in three technical replicates. Each bar represents the mean of three replicates (n = 3) ± SD. Data with different letters are considered significantly different (*p* ≤ 0.05).

The expressions of 18 SAP family genes after different treatments indicated that *OsSAP1* (LOC_Os09g31200), *OsSAP4* (LOC_Os02g10200), *OsSAP5* (LOC_Os02g32840), *OsSAP6* (LOC_Os03g57890), *OsSAP8* (LOC_Os06g41010), *OsSAP9* (LOC_Os07g07350), *OsSAP11* (LOC_Os08g39450), and *OsSAP17* (LOC_Os09g21710) had relatively higher expressions in the early development of rice seeds under normal conditions; meanwhile, they responded to high temperature and SPD induction. As shown in Figure 2B,C, the expression levels of those five *SAP* genes were significantly changed (Change more than 2 times) after HT treatment, and the expression of those five genes increased obviously after Spd pre-treatment. Among them, the expression level of *OsSAP5* was higher in seeds at 11 DAP, and the increase was most obvious after SPD induction. This suggested that *OSAP5* may be mainly involved in Spd-mediated enhancement of high-temperature resistance during rice seed development.

### 3.3. Over-Expression of OsSAP5 Enhanced the Heat Resistance of Arabidopsis Thaliana Seeds during Development

**Figure 3:**
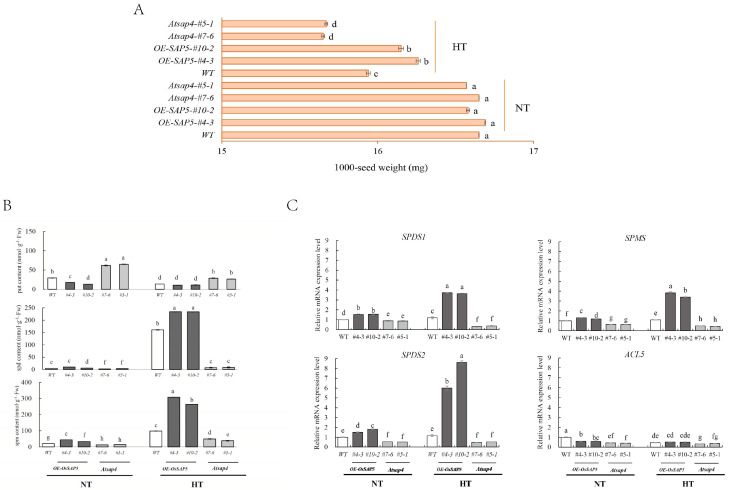
Effects of high temperature (35 °C, 4 h) during seed filling stage on 1000-seed weight and endogenous polyamines contents in harvest seeds of *Arabidopsis thaliana.* (**A**) The 1000-seed weight of *OE-OsSAP5 and Atsap4*. (**B**) Endogenous polyamine content of harvest seeds. (**C**) Relative mRNA expression level of polyamine synthesis related gene in harvest seeds. High-temperature treatment (HT, 35 °C for 4 h), normal temperature treatment (NT, 23 °C for 4 h). The bars mean standard deviation and different letters indicated significant differences between treatments at the same developmental stage (α = 0.05, LSD).

*OsSAP5* was genetically transformed into *Arabidopsis thaliana* to preliminarily verify its biological function in regulating grain filling heat resistance. Two homozygous lines of *OE-OsSAP5* were obtained and two mutant lines of *Atsap4* (homologous gene with *Ossap5* in *Arabidopsis thaliana*) were used as a control in this study. Under normal development conditions of 23°C (NT), the seedlings of wild-type (WT), *Atsap4,* and *OE*-*SAP5* grew normally, and there was no significant difference in the 1000-seed weight of mature seeds among all lines. However, at the early stage of seed development, high-temperature treatment significantly reduced the 1000-seed weight of all lines. Notably, the 1000-seed weight of *Atsap4* was significantly lower than that of WT, while *OE*-*SAP5* showed a significantly higher 1000-seed weight than WT (Figure 3A).

Under NT, the contents of Spd and Spm in all lines were low. The high-temperature at an early stage of seed development significantly increased the contents of Spd and Spm in WT seeds; meanwhile, as compared with WT, the contents of Spd and Spm in *OE-SAP5* and expressions of their related synthetic genes *AtSPDS1*, *AtSPDS2*, and *AtSPMS* were further increased. However, those changes were contrary in *Atsap4*, especially as the content of Spd in *AtSAP4* was significantly lower than that of WT and *OE-SAP5.* In addition, the Put content of *AtSAP4* was significantly higher than that of WT and *OE-SAP5* under NT. High temperature reduced the Put content of all strains, but the Put content of *AtSAP4* was still high (Figure 3B,C).

### 3.4. Spd Ameliorated the Heat Damage during the Germination Period of OE-SAP5 and Atsap4 

**Figure 4:**
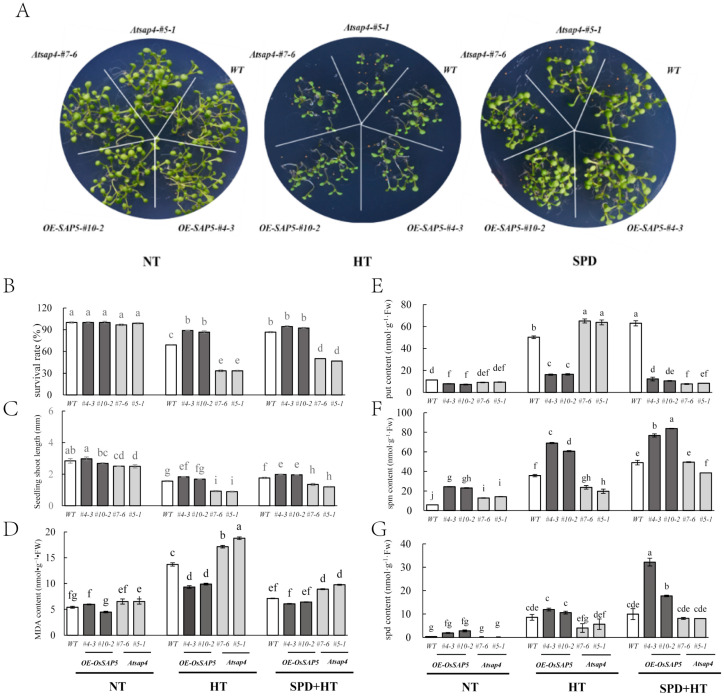
Effect of exogenous Spd application on seedling growth of different *Arabidopsis thaliana* under high-temperature stress during germination. (**A**) *Arabidopsis* seedling growth of WT, *OE*-*SAP5* and *Atsap4* under normal temperature conditions (NT, 23 °C), high-temperature stress (HT, 50 °C), and 1mM Spd was added to half-strength MS medium + high-temperature stress (SPD + HT). (**B**) Survival rate of *Arabidopsis* seedling. (**C**) Seedling shoot length. (**D**) The MDA content of *Arabidopsis* seedling. (**E**–**G**) The content of endogenous Put, Spd and Spm in *Arabidopsis* seedlings. Seeds of *Arabidopsis* (WT, *OE*-*SAP5* and *Atsap4*) were stratified at 4 °C for 2 days and then transferred to NT or HT or SPD + HT for another 7 h in dark. The bars mean standard deviation and different letters indicated significant differences between treatments at the same developmental stage (α = 0.05, LSD).

The seeds of WT, O*E-SAP5,* and *Atsap4* harvested under NT were used to test their germination performance under high temperatures. The results showed that the seeds of all lines germinated well and the seedlings grew normally under 23 °C. However, under high temperatures, seedling survival rate and seedling length decreased obviously to varying degrees. The seedling survival rate for O*E-SAP5* remained at 90%, compared with 65% for WT and only 30% for *Atsap4*. Similarly, O*E-SAP5* and WT had significantly higher seedling length than that of mutant. On the contrary, the MDA content of *Atsap4* was notably higher than O*E-SAP5* and WT. After Spd pre-spraying, the survival rate and seedling length of both WT and mutants were significantly increased under high temperature, while the MDA content changed in the opposite way (Figure 4A–D).

In comparison with WT, the endogenous Spd and Spm contents in *OE-SAP5* were significantly upregulated, while in the *Atsap4* plants they were significantly downregulated. High temperature-induced an increase in three polyamines in seedlings, the Spd and Spm contents in *OE-SAP5* were always significantly higher than those in WT and mutants, while the content of Put in *OE-SAP5* was significantly lower than those in WT and mutants. Spd pre-application further increased the Spd and Spm contents in Arabidopsis seedlings under high temperature, especially the Spm contents of WT and *Atsap4* were significantly higher than those without Spd application (Figure 4E–G).

## 4. Discussion

MDA content was positively correlated with fat oxidation [38], and was used as the candidate indicator for seed vigor evaluation. It was proposed that Spd played a key role in ROS scavenging by protected cell membrane stability and maintained organelle integrity under stress conditions [39]. For instance, it had been reported in the literature that PAs could be used as direct free radical scavengers or combined with antioxidant enzyme molecules to directly or indirectly scavenge free radicals in cells to reduce the oxidative damage received by plants [12,40]. Zhang [41] found that leaves sprayed with 1 mM Spd after flowering enhanced the heat tolerance by maintaining cell membrane stability, increasing antioxidant enzymes activities, and decreasing MDA content in fall fescue. Wi [42] found that the expression of antioxidant enzymes could be significantly induced by increasing PAs content in tobacco, thus inducing the tolerance of tobacco to different abiotic stresses. Under salt stress, exogenous Spd led to a significant decrease in MDA content and O_2_^-^ production rate [43,44]. Similarly, our results showed that under high-temperature stress, compared with WT, MDA content of *OE*-*SAP5 Arabidopsis* decreased while *Atsap4* was the opposite (Figure 4). Exogenous application of Spd improved the high temperature resistance of rice by increasing the POD activity in rice and reducing the MDA content in both rice and *Arabidopsis* (Figure 1 and Figure 4).

PAs had a positive effect on seed quality [45]. Huang [12] revealed that Spd promotes rapid seed germination and high seed vigor of sweet corn, which might be closely related to the metabolism of gibberellins, ABA, and ethylene. PAs played fundamental roles not only in normal growth and development but also in self-defense against heat stress. The content of endogenous PAs in the heat-resistant rice lines was more stable than that in the heat-sensitive lines under high-temperature stress [46]. Sagor [8] found that exogenously applied Spm had a potential to protect *Arabidopsis* plants from heat shock-induced damage, and the higher the Spm content the higher the thermotolerance. Goyal [47] found that winter wheat responds to the heat damage by increasing endogenous spd and spm and reducing put content. In this article, the different of put content in rice seeds (Figure 1C), and *Arabidopsis* seedlings (Figure 4E), after the application of Spd may be caused by differences in plant species and plant morphology. In addition, HT increased not only the expression levels of *OsADC1* but also *OsSPDS1* and *OsSPDS3* in young spikelets of heat-sensitive cultivars; however, HT exerted little influence on *OsODC1* and *OsSAMDC2*, ultimately increasing the levels of endogenous Spd and Spm [11]. In the present study, the exogenous application of Spd significantly increased the accumulation of endogenous Spd and Spm, which had the potential to protect rice (Figure 1C), and *Arabidopsis* (Figure 4E-G), from heat stress. However, the endogenous spd content of the wild type did not significantly increase after spraying of spd under heat stress (Figure 4G). We supposed that most of the endogenous spd of the plant has been converted to spm. While, the endogenous spd in *OE-OsSAP5* was significantly increased, *Ossap5* overexpression may cause the high expression of spd related genes, they promote and influence each other. Moreover, three genes (*AtSPDS1*, *AtSPDS2*, and *AtSPMS*) were upregulated against heat stress, might also indirectly contribute to the synthesis of Spd and Spm (Figure 3C). Those results indicated that exogenous Spd had a positive effect on improving plant heat tolerance. 

SAPs played important roles in plants abiotic stress responses. Studies had shown that AtSAP5 has E3 ubiquitin ligase activity and was involved in the regulation of heat-responsive genes [22]. Proteins harboring a RING domain were believed to play a role as ubiquitin ligases (E3) for the recognition and ubiquitylation of substrate proteins [48]. TaSAP5 was also reported as a RING E3 ubiquitin ligase, could identify and degrade TaDRIP (DREB2A INTERACTING PROTEIN), and lent to the accumulation of DREB2A (DEHYDRATION-RESPONSIVE ELEMENT BINDING PROTEIN2A) and the transcription of its downstream genes, thereby enhanced the drought tolerance of wheat. Zhang [49] further identified that HSP90 (CHLOROPLAST HEAT SHOCK PROTEIN 90) was the substrate protein of TaSAP5, and its degradation was mediated by 26S proteasome. Treatment of rice seedlings with GDA (geldamycin, HSP90 inhibitor) significantly increased the expression of the endogenous heat shock transcription factor *OsHsfA2* and the heat tolerance of the seedlings [50]. The role of HSFs in heat-tolerance and other abiotic stresses had been well confirmed [51], among which HsfA1s was the main regulator of the heat stress response [52]. It can upregulate the transcription levels of more than 200 heat-responsive genes, including *HSFA2*, *HSFA7a/7b*, and *DREB2A* [53,54]. In the *Arabidopsis* HSF family, DREB2A specifically induced the transcriptional expression of HsfA3, and HsfA3 also regulated the expression of HSP-encoding genes [55]. The latest study found that OsHSFA3 can bind to the promoters of *AtADC1* and *OsADC* which can increase the polyamine content in the plant and the expression levels of *AtADC1*, *AtADC2*, *SPDS1,* and *SPMS*, maintained the homeostasis of active oxygen and enhanced the drought tolerance of plant [56]. In our study, rice seeds harvested at the early grain-filling periods (11 DAP, after high temperature treatment) were used for transcriptome (mRNA) sequencing analysis to preliminarily screen the key genes regulated by Spd (data not shown). Then the gene expression profiling analysis of SAP family was further carried out. The results showed that eight *OsSAPs* had relatively high expression under normal conditions, which suggested that these genes may be closely related to seed development (Figure 2B) and there are five *OsSAPs* may strongly linked to heat stress (Figure 2C). Moreover, *OsSAP5* was the most upregulated expression induced by Spd, which was speculated that *OsSAP5* may be mainly involved in the Spd-mediated enhancement of high-temperature resistance during rice seed development. 

## 5. Conclusions

**Figure 5:**
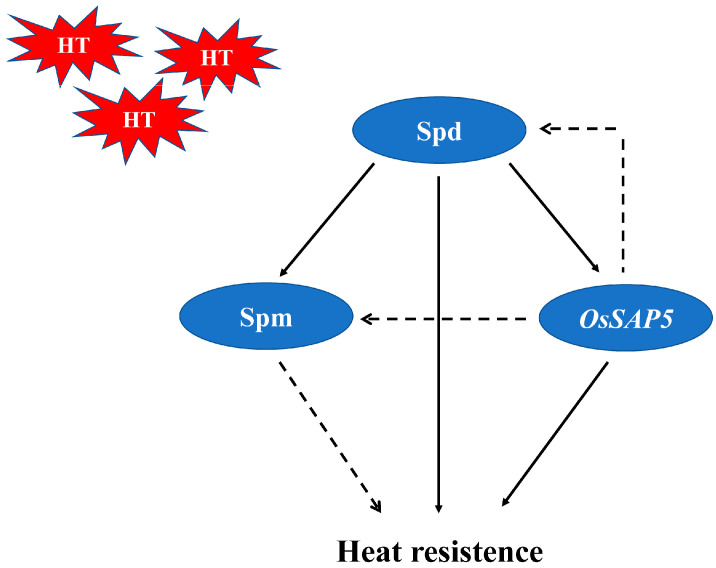
Hypothetical model of the role of PAs and *OsSAP5* under heat stress in rice. Exogenous Spd increased endogenous Spd content, and then upregulate the content of Spm, leading to an increased in heat resistance. *OsSAP5* was induced by Spd and may be involved in the rice heat resistance in coordination with Spd and Spm, and *OsSAP5* may also be able to affect the accumulation of spd. HT meant high temperature.

In this study, exogenous Spd alleviated the effect of heat damage on seed quality at grain filling stage and the damage of heat damage on seed germination at imbibition stage by increasing the contents of endogenous Spd and Spm and enhancing antioxidant enzyme activity. Meanwhile, *OsSAP5*, a key gene induced by Spd, might be involved in the rice heat resistance and seed quality in coordination with Spd and Spm (Figure 5). However, the regulatory mechanism between Spd and OsSAP5 still needs to be further explored.

## Figures and Tables

**Table 1:** Effects of Spd spraying after pollination on the quality of mature rice seeds which experienced high temperature during the early grain-filling stage.

Temperature	Treatment	GP (%)	GI	VI	1000 Grain Weight (g)
NT	H_2_O	85 ± 1.8 a	6.45 ± 0.32 a	0.64 ± 0.031 a	21.23 ± 0.04 a
HT	H_2_O	59 ± 0.7 b	3.82 ± 0.09 b	0.20 ± 0.011 c	16.78 ± 0.03 c
	Spd	87 ± 0.7 a	6.06 ± 0.12 a	0.38 ± 0.006 b	19.38 ± 0.08 b
	CHA	43 ± 0.7 c	2.48 ± 0.15 c	0.11 ± 0.008 d	16.08 ± 0.01 d

NT is a normal temperature of 26 °C 12 h light/21 °C 12 h dark, HT is a high-temperature of 40 °C 12 h light/35 °C 12 h dark, continuous treatment for 5 days. Spd: the treatment of spermidine spraying on the panicle after 3 days after pollination; CHA: cyclohexylamine spraying; H_2_O: the control sprayed with distilled water; GP: germination percentage; GI: germination index; VI: vigor index. Four biological replicates for each treatment were set in seed germination and seedling emergence tests. The lowercase letter after the number was the significant difference between treatments (LSD, α = 0.05).

## Data Availability

The data presented in this study are available within the article.

## Supplementary Materials

The following are available online at https://www.mdpi.com/article/10.3390/antiox10101544/s1, Table S1: Primers used in this study.
