# Supplementary material for: Spermidine Induces Expression of Stress Associated Proteins (SAPs) Genes and Protects Rice Seed from Heat Stress-Induced Damage during Grain-Filling
Source: Antioxidants (Basel). 2021 Sep 28;10(10):1544. doi: 10.3390/antiox10101544 (PMC8533277; doi:10.3390/antiox10101544)
Supplement: Supplementary file 1 [file antioxidants-10-01544-s001.zip › antioxidants-1373250-supplementary.pdf]

antioxidants

Table S1. Primers used in this study.

Name	Accession number	Purpose	Forward Primer (5′-3′)	Reverse Primer (5′-3′)
OsSAP1	LOC_Os09g31200	qRT-PCR	TTTTAATTGCAAACGGGAGGATA	TCGATTCTTTTTCCCTCAACCA
OsSAP2	LOC_Os01g52030	qRT-PCR	ACTGCTCGATCTTGCCAACA	GCAAATCAGCAATCGACCAA
OsSAP3	LOC_Os01g56040	qRT-PCR	GCGCAGTTACGTCTGATTCCT	CCGGCTCAGGGTTGTTCTT
OsSAP4	LOC_Os02g10200	qRT-PCR	TGGCAGCCGAAAGAATTCA	AACCACCAAGAATAAAGATTACACAACA
OsSAP5	LOC_Os02g32840	qRT-PCR	ACGCTCGACCTCTGCTCCAA	GATGGCGAGGAAGGATGAGT
OsSAP6	LOC_Os03g57890	qRT-PCR	GGAGGCACCTTCTGTTCAATG	CTCCCGACCTGCTTGTAATCA
OsSAP7	LOC_Os03g57900	qRT-PCR	GAGGCCACCAACAACATGTG	CTCCACGGCGTCGTTGTC
OsSAP8	LOC_Os06g41010	qRT-PCR	TCTCTGGTCAGTCCGAATGGT	TCAGCGCAAGACGATCATAAA
OsSAP9	LOC_Os07g07350	qRT-PCR	CGACTGCACCTTCGACTACAAG	GGCTATCACAACAGGATTCTGCTT
OsSAP10	LOC_Os07g07400	qRT-PCR	CCGGGGACAGTGACAAAACATC	CATCACCGTCGTCCCCTCGCCT
OsSAP11	LOC_Os08g39450	qRT-PCR	ACGACTGCAGCTTCGACTACAA	TGTATTATCCTTTTAGAACCTAACGATCTT
OsSAP12	LOC_Os08g33880	qRT-PCR	CGCCTGCACCTTCGACTT	CGATCAGCGGGTTCTCCTT
OsSAP13	LOC_Os01g51990	qRT-PCR	TTTATTCACGTGCTCGGTTAGGAT	AAATGTCGTTGGCGTTTCGTA
OsSAP14	LOC_Os03g57920	qRT-PCR	AAGCTGGCCTTCAGGATTTG	TTGATCGGCTACTTCGATTTGA
OsSAP15	LOC_Os05g23470	qRT-PCR	GCAGTCTACATCAAGCGAAAAATC	AGGCGGACGATGAACAAGAG
OsSAP16	LOC_Os07g38240	qRT-PCR	CATTGCGAAAAGTCCCATCA	GGCAGACATCGACCGTCACT
OsSAP17	LOC_Os09g21710	qRT-PCR	GGAGGCATTTGATCTGTCCAA	AACCATTTCCCCTGTTAAGAATAGC
OsSAP18	LOC_Os07g07370	qRT-PCR	CACCGGGAACGGCGACAAAGTGGT	CCGAGAGGTCGGCGACGACCTTG
AtSPDS1	LOC_At1g23820	qRT-PCR	GCAATAACCATGGACGCTAAA	CCTCTTCTCTCGGTCTTTTCAA
AtSPDS2	LOC_At1g70310	qRT-PCR	CAACCCCAAAAAGGTACTGG	GCCACTTCCCTCAGGACTC
AtSPMS	LOC_At5g53120	qRT-PCR	CCCCTAAAAATGTAATTTCTCACC	TTGCAACAGGCTACAAGATATAGG
ATACL5	LOC_At5g19530	qRT-PCR	TGTTTATAATGGGAGGAGGTGAA	TTCTCGATCGTCGTGTGTTT
OsActin	LOC_Os03g50885	qRT-PCR	CTTCATAGGAATGGAAGCTGCGGGT	CGACCACCTTGATCTTCATGCTGCT
